# Supplementary material for: Iron Acquisition of Urinary Tract Infection Escherichia coli Involves Pathogenicity in Caenorhabditis elegans
Source: Microorganisms. 2021 Feb 2;9(2):310. doi: 10.3390/microorganisms9020310 (PMC7913171; doi:10.3390/microorganisms9020310)
Supplement: Supplementary file 1 [file microorganisms-09-00310-s001.pdf]

Supplementary information

Microorganisms
Research article

Iron acquisition of urinary tract infection *Escherichia coli* involves pathogenicity in *Caenorhabditis elegans*

(Pathogenicity of UTI *E. coli* in *C. elegans*)

Masayuki Hashimoto[1,2,3*], Yi-Fen Ma[1], Sin-Tian Wang[3,4], Chang-Shi Chen[3,4] and Ching-Hao Teng[1,2,3*]

[1] Institute of Molecular Medicine, College of Medicine, National Cheng Kung University, Tainan, Taiwan (ROC).
[2] Center of Infectious Disease and Signaling Research, National Cheng Kung University, Tainan, Taiwan (ROC).
[3] Institute of Basic Medical Sciences, College of Medicine, National Cheng Kung University, Tainan, Taiwan (ROC)
[4] Department of Biochemistry and Molecular Biology, College of Medicine, National Cheng Kung University, Tainan, Taiwan (ROC).

Fig S1. The pathogenicity of *E. coli* clinical isolates in different symptoms and phylogenetic groups.
Fig S2. The number of virulence factors in clinically isolated *E. coli*.
Table S1. Primers used in this study.
Table S2. *E. coli* strains used for pathogenic solid assay
Table S3. Symptoms of clinically isolated 133 *E. coli* strains

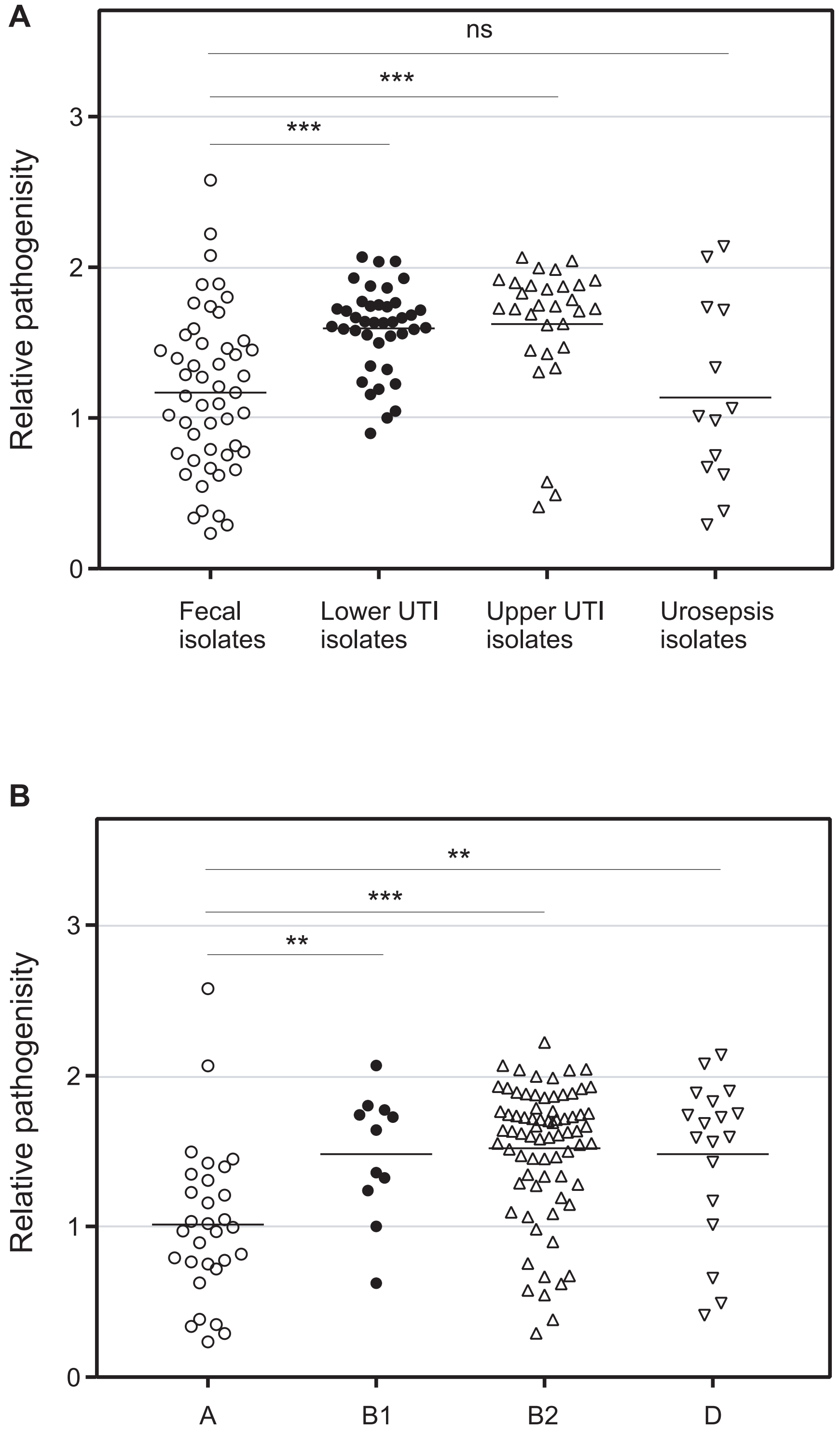

Fig S1. The pathogenicity of *E. coli* clinical isolates in different symptoms and phylogenetic groups. Pathogenicity (relative OD_{592nm} value in liquid assay) of the 133 stains were plotted in different symptoms (panel A) and phylogenetic groups (panel B). The horizontal bars represented the mean values. The results were analyzed statically by Mann-Whitney U test (**, p<0.01; ***, p<0.001; ns, not significant).

A

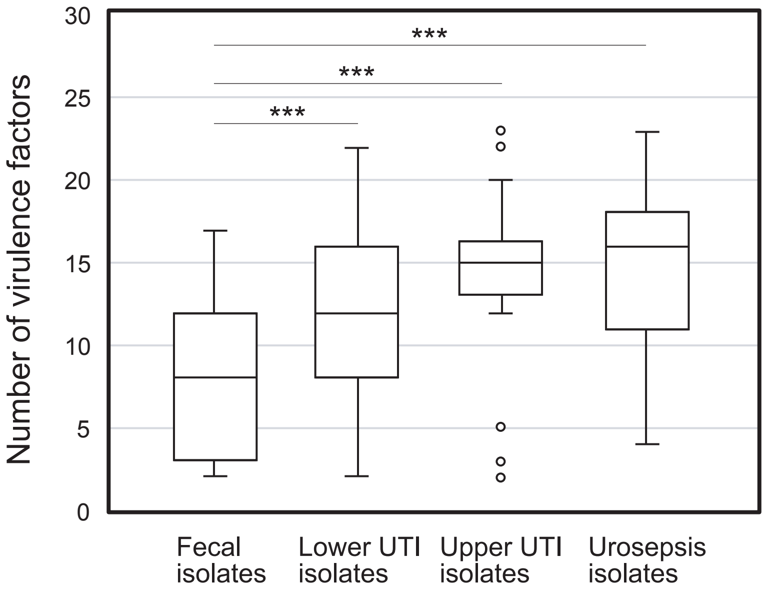

B

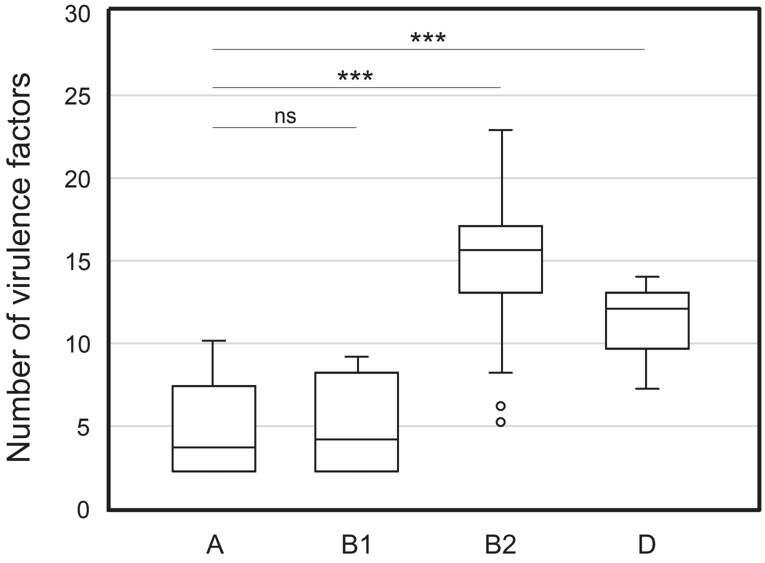

Fig S2. Number of virulence factors in clinically isolated *E. coli*. Box-plot indicates the number of VFs in each symptom (panel A) and in each phylogenetic group (panel B). The data was statically analyzed by Mann-Whitney U test (***, $p<0.001$; ns, not significant different).

Table S1. Primers used in this study.

**Phylogenetic analysis**			
	**Target**	**Primer**	**Sequence**
	arpA	AceK.f	AACGCTATTCGCCAGCTTGC
		ArpA1.r	TCTCCCCATACCGTACGCTA
	chuA	chuA.1b	ATGGTACCGGACGAACCAAC
		chuA.2	TGCCGCCAGTACCAAAGACA
	yjaA	yjaA.1b	CAAACGTGAAGTGTCAGGAG
		yjaA.2b	AATGCGTTCCTCAACCTGTG
	TspE4C2	TspE4C2.1b	CACTATTCGTAAGGTCATCC
		TspE4C2.2b	AGTTTATCGCTGCGGGTCGC
	arpA	ArpAgpE.f	GATTCCATCTTGTCAAAATATGCC
		ArpAgpE.r	GAAAAGAAAAAGAATTCCCAAGAG
	trpA	trpAgpC.1	AGTTTTATGCCCAGTGCGAG
		trpAgpC.2	TCTGCGCCGGTCACGCCC
**Virulence factor**			
	**Target**	**Primer**	**Sequence**
	sit A	sitA-F	CGCAGGGGGCACAACTGAT
		sitA-R	CCCTGTACCAGCGTACTGG
	cvaC	cvaC-F	CACACACAAACGGGAGCTGTT
		cvaC-R	CTTCCCGCAGCATAGTTCCAT
	bmaE	bmaE-F	ATGGCGCTAACTTGCCATGCTG
		bmaE-R	AGGGGGACATATAGCCCCCTTC
	fimH	fimH-F	TCGAGAACGGATAAGCCGTGG
		fimH-R	GCAGTCACCTGCCCTCCGGTA
	focH	focH-F	ATGCGTAAATATTATCCCCTC
		focH-R	ACCTGTCTGGATATAGAC
	sfaH	sfaH-F	AACCGTCACCTATACCTATG
		sfaH-R	TGCCAGTAAATCTCCGTTGT
	feoB	feoB-F	AATTGGCGTGCATGAAGATAACTG
		feoB-R	AGCTGGCGACCTGATAGAACAATG
	iucC	iucC-F	CGCCGTGGCTGGGGTAAG
		iucC-R	CAGCCGGTTCACCAAGTATCACTG
	vat	vat-F	AGAGACGAGACTGTATTTGC
		vat-R	GTCAGGTCAGTAACGAGCAC
	picU	picU-F	GGGTATTGTCCGTTCCGAT
		picU-R	ACAACGATACCGTCTCCCG
	malX	malX-F	GGACATCCTGTTACAGCGCGCA
		malX-R	TCGCCACCAATCACAGCCGAAC
	fyuA	fyuA-F	TGATTAACCCCGCGACGGGAA
		fyuA-R	CGCAGTAGGCACGATGTTGTA
	tsh	tsh-F	CCGTACACAAATACGACGG
		tsh-R	GGATGCCCCTGCAGCGT
	cdtB	cdtB-F	GAAAATAAATGGAACACACATGTCCG
		cdtB-R	AAATCTCCTGCAATCATCCAGTTA
	hek	hek-F	CGAATCGTTGTCACGTTCAG
		hek-R	TATTTATCGCCCCACTCGTC
	irp2	irp2-F	AAGGATTCGCTGTTACCGGAC
		irp2-R	TCGTCGGGCAGCGTTTCTTCT
	iutA	iutA-F	GGCTGGACATCATGGGAACTGG
		iutA-R	CGTCGGGAACGGGTAGAATCG
	traT	traT-F	GGTGTGGTGCGATGAGCACAG
		traT-R	CACGGTTCAGCCATCCCTGAG
	draB/afa	Afa-F	GGCAGAGGGCCGGCAACAGGC
		Afa-R	CCCGTAACGCGCCAGCATCTC
	chuA	chuA-F	ATGGTACCGGACGAACCAAC
		chuA-R	TGCCGCCAGTACCAAAGACA
	ibeA	ibe10-F	AGGCAGGTGTGCGCCGCGTAC
		ibe10-R	TGGTGCTCCGGCAAACCATGC
	ompT	OMPT-F	ATCTAGCCGAAGAAGGAGGC
		OMPT-R	CCCGGGTCATAGTGTTCATC
	iha	IHA-F	CTGGCGGAGGCTCTGAGATCA
		IHA-R	TCCTTAAGCTCCCGCGGCTGA
	papG II	Allele II-F	GGGATGAGCGGGCCTTTGAT
		Allele II-R	CGGGCCCCCAAGTAACTCG
	papG III	Allele III-[illegible]	GGCCTGCAATGGATTTACCTGG
		Allele III-[illegible]	CCACCAAATGACCATGCCAGAC
	cnf1	cnf1-F	AAGATGGAGTTTCCTATGCAGGAG
		cnf2-R	CATTCAGAGTCCTGCCCTCATTATT
	hlyA	hly-F	AACAAGGATAAGCACTGTTCTGGCT
		hly-R	ACCATATAAGCGGTCATTCCCGTCA
	sat	sat-F	GCAGCTACCGCAATAGGAGGT
		sat-R	CATTCAGAGTACCGGGGCCTA
	ireA	IRE-F	GATGACTCAGCCACGGGTAA
		IRE-R	CCAGGACTCACCTCACGAAT
	iroN	IRONEC-F	AAGTCAAAGCAGGGGTTGCCCG
		IRONEC-R	GACGCCGACATTAAGACGCAG
	usp	usp-F	ACATTCACGGCAAGCCTCAG
		usp-R	AGCGAGTTCCTGGTGAAAGC
**Mutant construction**			
		**Primer**	**Sequence**
		chuA-P1	GAGAATCGCTATGTCACGTCCGCAATTTACCTCGTTGCGTGTGTAGGCTGGAGCTGCTTC
		chuA-P2	TAACTCACGAAAATTTTTCCGTTACGACCATCCTGTGGGAATGGGAATTAGCCATGGTCC
		fyuA-P1	GGACTCACAACAATGAAAATGACACGGCTTTATCCTCTGGGTGTAGGCTGGAGCTGCTTC
		fyuA-P2	ATAATCAGAAGAAATCAATTCGCGTATTGATACCGACGGTATGGGAATTAGCCATGGTCC
		165-1	AGCAAAAGGTGGTGGATCGC
		165-2	GAGAGCACTGTCCAGACGAG

Table S2. E. coli strains used for pathogenic solid assay

Strain	Genotype	Source
E. coli UTI89	wold type	Laboratory stock
MH819	E. coli UTI89 ΔchuA::Km	This study
MH820	E. coli UTI89 ΔfyuA::Cm	This study
MH822	E. coli UTI89 ΔentA::Km	This study
MH821	E. coli UTI89 ΔchuA::scar ΔfyuA::scar	This study
MH823	E. coli UTI89 ΔchuA::scar ΔfyuA::scar ΔentA::Km	This study

Table S3. Symptoms of clinically isolated 133 *E. coli* strains

Symptoms	Number of strains
Fecal isolates	50 [a]
Lower UTI isolates	40 [b]
Upper UTI isolates	30
Urosepsis	13 [c]

a, including K-12 MG1655

b, including UTI89

c, including CFT073
